# Intergenerational Socioeconomic Mobility and Cardiovascular Health Among Hispanic/Latino Youth and Caregivers Living in the United States

**DOI:** 10.1161/JAHA.125.042972

**Published:** 2025-11-26

**Authors:** Monica Batalha, Paola Filigrana, Krista M. Perreira, Amanda C. McClain, Amber Pirzada, Martha Daviglus, Christina Cordero, Alan M. Delamater, Linda C. Gallo, Carmen R. Isasi

**Affiliations:** ^1^ Department of Epidemiology and Population Health Albert Einstein College of Medicine Bronx New York; ^2^ Department of Social Medicine University of North Carolina School of Medicine Chapel Hill North Carolina; ^3^ School of Exercise and Nutritional Sciences San Diego State University San Diego California; ^4^ Institute for Minority Health Research University of Illinois at Chicago Chicago Illinois; ^5^ Department of Psychology University of Miami Miami Florida; ^6^ Mailman Center for Child Development University of Miami Miami Florida; ^7^ Department of Psychology San Diego State University San Diego California

**Keywords:** cardiovascular health, caregiver, Hispanic or Latino, socioeconomic mobility, youth, Epidemiology, Cardiovascular Disease, Lifestyle, Obesity

## Abstract

**Background:**

Little is known about the effects of socioeconomic mobility on cardiovascular health (CVH) across the generations. We evaluated the association between caregiver intergenerational socioeconomic mobility and caregivers’ and youth’s CVH. We also explored whether the association was modified by the youth’s sex and age.

**Methods:**

We used data from the HCHS/SOL (Hispanic Community Health Study/Study of Latinos) (2008–2011) and its ancillary study SOL Youth (Hispanic Community Children’s Health Study/Study of Latino Youth) (2012–2014). Socioeconomic mobility was assessed using the grandparents’ and caregivers’ socioeconomic position (SEP) and was classified as stable low, downward, upward, and stable high groups. CVH was assessed based on the American Heart Association Life’s Essential 8. Survey linear regression models were used to investigate the association between socioeconomic mobility and caregivers’ and youth’s CVH, adjusting for age, sex, place of birth, Hispanic/Latino background, and field center.

**Results:**

Caregivers with lower CVH scores were observed in stable low (β=−3.91 [95% CI, −7.33 to −0.49]) and downward (β=−4.85 [95% CI, −9.61 to −0.09]) SEP groups compared with those in the stable high group. Caregivers with lower body mass index scores were observed in the stable low (β=−7.87 [95% CI, −15.31 to −0.43]) versus stable high SEP group. We found a lower total CVH (β=−3.00 [95% CI, −5.24 to −0.75]) score in youth whose caregivers were in the stable low SEP group versus those whose caregivers were in the stable high SEP group, and this result was modified by the youth’s sex and age.

**Conclusion:**

Among Hispanic/Latino youth and their caregivers, stable low SEP was associated with worse CVH versus stable high SEP.

**Nonstandard Abbreviations and Acronyms**
Add Health: National Longitudinal Study of Adolescent to Adult Health
AHA: American Heart Association
CVH: cardiovascular health
DOHaD: Developmental Origins of Health and Disease
FHS: Framingham Heart Study
GPAQ: Global Physical Activity Questionnaire
HCHS/SOL: Hispanic Community Health Study/Study of Latinos
HEI 2010: Healthy Eating Index 2010
HELENA: Healthy Lifestyle in Europe by Nutrition in Adolescence
LE8: Life’s Essential 8
LS7: Life’s Simple 7
MINDUS: Maternal and Infant Neurodevelopment Study
NHANES: National Health and Nutrition Examination Survey
SEP: socioeconomic position
SOL Youth: Hispanic Community Children’s Health Study/Study of Latino Youth

**Research Perspective**

**What Is New?**

- Among Hispanic/Latino caregivers and youth, socioeconomic mobility was associated with cardiovascular health. The intergenerational persistence of lower socioeconomic position was associated with worse cardiovascular health in youth and their caregivers compared with the intergenerational persistence of higher socioeconomic position.

**What Question Should Be Addressed Next?**

- Future research should focus on understanding the critical periods (age stages during the lifespan) during which structural interventions can have a lasting impact in preventing adverse cardiovascular health outcomes.

Social determinants play an important role in cardiovascular health (CVH).^1^, ^2^ Previous studies have shown that the association between socioeconomic position (SEP) and CVH varies by race and ethnicity and may be modified by sex.^3^, ^4^, ^5^ In addition, individuals with higher levels of education were more likely to have better CVH, as measured using the American Heart Association (AHA) Life’s Simple 7 (LS7), and those with lower levels of education were associated with lifetime cardiovascular disease risk.^2^, ^6^, ^7^ Data from the National Health and Nutrition Examination Survey (NHANES), a national survey in the United States, have shown that immigrants who were male, Hispanic, and living for ≥15 years in the United States had lower CVH, as measured using AHA Life’s Essential 8 (LE8).^8^ The main determinants of worse CVH among US immigrants included household food insecurity, lower education, and reduced income levels.^8^ This is consistent with previous studies showing that socioeconomic disadvantage is a leading stressor and risk factor for suboptimal CVH among the Hispanic/Latino population living in the United States.^9^, ^10^

From the life course perspective, socioeconomic mobility is often described as a change in people’s SEP throughout their life course (intragenerational mobility) or between ≥2 generations of SEP (intergenerational mobility).^11^, ^12^ Previous studies using HCHS/SOL (Hispanic Community Health Study/Study of Latinos) evaluated how socioeconomic mobility and subjective social status were related to CVH.^9^, ^13^ An increase in subjective social status was associated with a higher overall CVH, measured using the AHA LS7.^13^ The other study showed that high childhood and adult SEP, socioeconomic mobility, and stable high SEP were associated with better CVH, measured using 4 health factors of the AHA LE8.^9^ However, none of these studies evaluated how socioeconomic disadvantage influences CVH across generations.

Expanding the research on the importance of family relations (eg, parent–child, grandparent‐grandchild) and the role of intergenerational transmission of SEP and health is paramount for understanding health inequalities.^14^, ^15^ Although the Hispanic/Latino populations have shown rates of intergenerational mobility similar to non‐Hispanic/Latino White populations, they have lower wages.^16^, ^17^ Therefore, there is still an intergenerational wealth gap between Hispanic/Latinos and non‐Hispanic/Latino White individuals.^16^, ^17^ In addition, there is evidence of a high prevalence of CVH risk factors, such as obesity, among the Hispanic/Latino population living in the United States.^10^, ^18^ In this context, we hypothesized that enduring socioeconomic disadvantages may be associated with worse CVH across generations. Thus, the main aim of this study was to evaluate the association of caregiver intergenerational socioeconomic mobility with caregivers’ and youth’s CVH, using the AHA LE8 and its metrics scores. The present study uses 3 generations (grandparents, caregivers, and youth) to address the research gap related to the long‐term health effects of the transmission of disadvantages on health. There is previous evidence regarding the differences that sex‐ and age‐related factors (eg, puberty) play in CVH risk and CVH inequality.^19^, ^20^, ^21^, ^22^, ^23^, ^24^, ^25^ Thus, we also investigated whether the associations of socioeconomic mobility and youth CVH changed according to youth sex and age and were independent of the caregiver’s CVH.

## METHODS

### Data Availability Statement

The data that support the findings of this study are available from the corresponding author upon reasonable request.

### Study Population and Design

**Figure 1:**
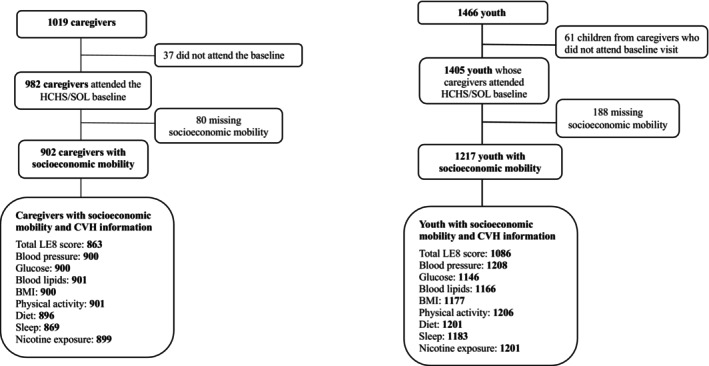
Flowchart of caregivers and youth participants in HCHS/SOL and SOL Youth before data set imputation. BMI indicates body mass index; CVH, cardiovascular health; HCHS/SOL, Hispanic Community Health Study/Study of Latinos; LE8, Life’s Essential 8; and SOL Youth, Hispanic Community Children’s Health Study/Study of Latino Youth.

This is a cross‐sectional study using data from HCHS/SOL and its ancillary study SOL Youth (Hispanic Community Children’s Health Study/Study of Latino Youth). HCHS/SOL is an ongoing, population‐based cohort study of 16 415 Hispanic/Latino adults that aims to identify the risk and protective factors for cardiovascular disease and other chronic conditions.^26^ From 2008 to 2011, HCHS/SOL recruited participants aged 18 to 74 years using a multistage probability sampling design from randomly sampled census block areas within the 4 field centers in the United States (Bronx, NY; Chicago, IL; Miami, FL; San Diego, CA).^26^, ^27^ Between 2012 and 2014, SOL Youth enrolled 1466 youth aged 8 to 16 years living in the household of a parent/caregiver who were participants in HCHS/SOL.^28^ We excluded caregivers (*n*=37) and youth (*n*=61) whose legal caregivers did not attend the baseline visit of HCHS/SOL (Figure 1). The institutional review boards of each corresponding site institution were responsible for approving HCHS/SOL and SOL Youth. A combination of written informed consent and assent was obtained from participants.

### Caregiver Socioeconomic Mobility

Caregiver intergenerational socioeconomic mobility was assessed using the grandparents’ and caregivers’ SEP information, self‐reported by caregivers at HCHS/SOL baseline (2008–2011). Grandparents’ SEP was defined according to the highest level of education attained by grandmother or grandfather: lower (<high school) or higher (≥high school). Caregivers’ SEP was defined according to education and income in adulthood. As the overall cohort participants are predominantly from low SEP, we used a low threshold to classify individuals as lower (<$30 000) or higher (≥$30 000) income, which more closely classifies individuals as being below or above the poverty line. Caregivers’ SEP was categorized as lower (education <high school and income <$30 000) or higher (education ≥high school or income ≥$30 000).

Caregiver socioeconomic mobility was classified into 4 categories: stable low (grandparents and caregivers with lower SEP), downward mobility (grandparents with higher SEP and caregivers with lower SEP), upward mobility (grandparents with lower SEP and caregivers with higher SEP), and stable high (grandparents and caregivers with higher SEP).

### Caregivers’ and Youth’s CVH

Our outcomes included the caregivers’ (collected at HCHS/SOL baseline) and youth’s (collected at SOL Youth) CVH, based on the AHA LE8 and its metrics. AHA LE8 is a construct to quantify CVH that contains 8 metrics, including 4 health factors (body mass index [BMI], blood lipids, blood glucose, and blood pressure) and 4 behavioral factors (diet, physical activity, nicotine exposure, and sleep).^29^ The total score (0–100 points) and each CVH metric score (0–100 points each) were used as the outcomes in our analyses. The total score was calculated as the unweighted average of all 8 metrics scores. A higher LE8 score represents better CVH. Details about how each component of the LE8 score was calculated can be found in Table S1, and a summary is provided below. For descriptive purposes only, we also classified the total score into high (≥80), moderate (≥50 to <80), and low (<50) CVH following the AHA’s recommendation.^29^

#### Body Mass Index

For caregivers, BMI was calculated as weight in kilograms divided by height in meters squared. For youth, the age‐sex‐specific BMI percentiles were generated according to the Centers for Disease Control and Prevention growth charts for children and adolescents.^30^

#### Laboratory Measures

All blood specimens were drawn in the morning under fasting conditions, processed on site, and stored at −70 °C. A central laboratory (University of Minnesota’s Advanced Research and Diagnostic Laboratory) performed all assays. Fasting glucose was measured using a hexokinase enzymatic method (Roche Diagnostics). Hemoglobin A_1c_ was measured from whole blood using high‐performance liquid chromatography using a Tosoh Automated HPLC Analyzer (Tosoh Bioscience, Inc). Total cholesterol was measured using a cholesterol oxidase enzymatic method (Roche Diagnostics), and high‐density lipoprotein cholesterol was measured using a direct magnesium/dextran sulfate method (Roche Diagnostics). Non–high‐density lipoprotein cholesterol was calculated as the difference between total cholesterol and high‐density lipoprotein cholesterol.

#### Blood Pressure

Three systolic and diastolic blood pressure measures were collected using an automatic sphygmomanometer after the participants rested for 5 minutes. The average of the 3 measures was used to calculate the blood pressure metric scores for youth and their caregivers. We generated age‐specific percentiles to classify the blood pressure metric of LE8 for participants younger than 13 years (Table S1).

#### Diet

Participants completed 2 interviewer‐administered 24‐hour diet recalls. We used the Healthy Eating Index 2010 (HEI 2010)^31^ to calculate the diet metric of LE8 for youth and their caregivers (Table S1).

#### Physical Activity

Self‐reported duration of total physical activity of the caregivers was determined using an adaptation of the World Health Organization Global Physical Activity Questionnaire (GPAQ).^32^, ^33^ Regarding youth, a 68‐item self‐reported questionnaire designed for SOL‐Youth was used to evaluate the amount of time per day spent engaging in moderate to vigorous activities in the past month.^34^ We calculated the physical activity score based on the LE8 recommendation for moderate and vigorous activity per week.^29^ For caregivers, each minute of moderate activity should count as 1 minute, and each minute of vigorous activity should count as 2 minutes for the total week. For the youth, each minute of activity (moderate or vigorous) should count as 1 minute.^29^

#### Sleep Health

A short self‐reported questionnaire was used to assess sleep duration during weekdays and weekends.^35^ We calculated the weekly average of sleep duration using the following formula: (weekday value×5+weekend value×2)/7.

#### Nicotine Exposure

To calculate the nicotine exposure metric, we used questionnaires to assess the use of tobacco or exposure to secondhand smoke by caregivers and youth.^36^ The use of lipid‐lowering or antihypertensive medications was collected from questionnaires.

### Covariates

The sociodemographic characteristics for youth and their caregivers included: age (continuous), sex (male, female), place of birth (including 50 states and District of Columbia: US‐born or US territory/foreign‐born), field center (Bronx, Chicago, Miami, and San Diego), and Hispanic/Latino background (Central American, Cuban, Dominican, Mexican, Puerto Rican, South American, and more than one/other). Caregivers also reported years living in the United States (continuous) and relationship with the youth (biological parent or other).

### Statistical Analysis

The HCHS/SOL sample design and cohort selection have been previously described.^27^ Briefly, a stratified 2‐stage area probability sample of household addresses was selected in each of the 4 field centers. The first sampling stage randomly selected census block groups with stratification based on Hispanic/Latino concentration and proportion of high/low socioeconomic status. The second sampling stage randomly selected households, with stratification, from the US Postal Service registries that covered the randomly selected census block groups. The sampling weights are the product of a “base weight” (reciprocal of the probability of selection) and 3 adjustments: (1) nonresponse adjustments made relative to the sampling frame; (2) trimming to handle extreme values (to avoid a few weights with extreme values being overly influential in the analyses); and (3) calibration of weights to the 2010 US Census according to age, sex, and Hispanic background. Descriptive statistics and regression models accounted for stratification and clustering by primary sampling units, weighted to adjust for sampling probability of selection and nonresponse with the use of complex survey procedures using the svy function in STATA, version 18 (StataCorp LLC).

Considering that 7.6%, 3.4%, and 5.8% of data on grandfather’s educational attainment, grandmother’s educational attainment, and caregiver’s household annual income, respectively, were missing, we conducted multiple imputations under the assumption that this information was missing at random. The imputation models included all variables used in the caregiver’s final model. We used sequential imputation and chained equations to create 50 imputed data sets using the mi function in STATA.^37^ For checking diagnostics and convergence, we created diagnostic plots and compared the distributions of observed and imputed values. In the pooling step and accounting for the within‐and‐between‐imputation variability, we used Rubin combination rules to generate the summary estimates and 95% CIs of the association.^37^

For the association of caregiver intergenerational socioeconomic mobility with both caregivers’ and youth’s CVH scores, we utilized survey linear regression models. Models were adjusted for age, sex, place of birth, Hispanic/Latino background, and field center, as there is previous literature evidence linking these demographic factors with both SEP and overall CVH and its metrics.^1^, ^23^, ^24^, ^28^, ^38^, ^39^, ^40^ In addition to the sampling weight, we adjusted our models for field center because people with specific Hispanic/Latino backgrounds tend to concentrate in specific geographic areas, which means that not all backgrounds were present in each study center. A *P* value ≤0.05 was considered statistically significant. We evaluated the normality of residuals and assessed homoscedasticity by plotting residuals against predicted values. Secondary analyses were performed with each continuous measure of LE8 health (BMI, blood lipids, blood glucose, and blood pressure) and behavioral (diet, physical activity, nicotine exposure, and sleep) factors as outcomes. In addition, we tested whether the association of socioeconomic mobility and youth’s CVH (and each metric) was independent of the caregiver’s health, adjusting our youth models for caregivers’ overall CVH score and each metric. We also tested sex and age interactions in the youth models. We dichotomized age as <12 years or ≥12 years to account for potential differences by pubertal status. We tested sex interactions based on previous literature evidence regarding the differences in CVH by sex.^23^, ^24^ As a sensitivity analysis, we performed the same models to test the association of life course socioeconomic mobility and CVH of parents and youth in a subset of only biological parents (≈95% of our sample), and we compared the estimates and CIs between this subset and the data set with all caregivers.

## RESULTS

Tables 1 and 2 show that the most reported Hispanic/Latino background was Mexican (caregiver: 49.8%; youth: 48.7%), and most participants were in the stable high (caregiver: 24.6%; youth: 37.2%) and upward (caregiver: 48.1%; youth: 33.6%) mobility groups. Table 1 shows that caregivers were predominantly foreign‐born (84.5%), female (88.9%), and biological parents (94.8%). Only 31.3% and 21.5% of caregivers and youth were classified in the high CVH category, respectively (Tables 1 and 2). Table 1 shows that the lowest mean±SD of total LE8 (67.6±16.1), diet (27.8±31.2), and nicotine exposure (51.5±47.7) scores were observed among caregivers in the downward mobility group. Children of caregivers in the stable low or downward groups had the lowest mean±SD of total LE8 (69.6±9.2 and 69.9±8.7), glucose (94.7±14.0 and 93.7±15.2), and nicotine exposure (83.5±27.1 and 84.6±23.8) scores, respectively (Table 2).

**Table 1:** Characteristics of Caregivers According to Intergenerational Socioeconomic Mobility: HCHS/SOL

Characteristics	Socioeconomic mobility*				Total
	Stable high	Stable low	Downward	Upward	
Percentage	24.6	18.3	9.0	48.1	100.0
Age, y	38.0 (0.6)	39.7 (0.8)	35.5 (0.9)	39.1 (0.4)	38.6 (0.3)
Sex, %					
Female	86.3	90.5	98.8	87.8	777(88.9)
Male	13.8	9.5	1.2	12.2	124(11.1)
Nativity, %					
Foreign‐born	77.7	93.3	70.5	87.2	773(84.5)
Born in the 50 US states	22.3	6.7	29.5	12.8	127(15.5)
Relationship with youth, %					
Biological parents	96.4	93.4	94.0	94.6	835(94.8)
Other	3.6	6.6	6.0	5.4	61(5.2)
Years living in the United States	19.5(1.0)	17.8(1.2)	20.1(1.9)	17.7(0.7)	18.4(0.5)
Hispanic background, %					
Central or South American	13.2	12.7	15.1	13.0	146 (13.2)
Cuban	11.0	1.6	7.7	5.9	73(6.6)
Dominican	17.0	7.0	16.1	16.9	117(15.1)
Mexican	35.1	68.5	24.9	54.9	436(49.8)
Puerto Rican	19.5	9.6	34.4	7.5	109(13.3)
More than 1/other	4.2	0.5	1.8	1.8	20(2.2)
Diet quality score (HEI‐2010)†	59.1(1.3)	62.4(1.2)	53.2(2.1)	62.5(0.9)	60.8(0.8)
Total score LE8, %					
Low CVH (<50)	7.6	8.1	12.9	4.6	63(6.7)
Moderate CVH (≥50 to <80)	63.2	66.0	58.1	60.7	548(62.0)
High CVH (≥80)	29.2	25.9	29.0	34.7	252(31.3)
Total score LE8‡	71.2(14.4)	70.3(13.0)	67.6(16.1)	73.1(12.6)	71.7(13.5)
Blood pressure score	82.9(24.3)	84.6(24.0)	86.3(22.2)	83.9(25.7)	84.0(24.7)
Blood glucose score	89.6(22.7)	81.7(30.5)	85.8(28.6)	87.1(24.5)	86.6(25.7)
Blood lipids score	67.2(29.9)	64.5(29.2)	76.2(28.0)	65.8(29.1)	66.8(29.3)
BMI score	54.9(35.0)	49.3(29.5)	48.1(37.1)	57.3(31.9)	54.4(32.9)
Physical activity score	73.2(41.8)	74.1(40.0)	74.9(40.3)	75.0(40.7)	74.4(40.7)
Diet score	40.6(33.3)	49.1(29.1)	27.8(31.2)	47.7(31.2)	44.5(31.9)
Sleep score	87.3(22.0)	84.4(23.3)	85.0(20.5)	88.5(19.9)	87.1(21.1)
Nicotine exposure score	74.7(39.1)	76.6(36.9)	51.5(47.7)	77.9(37.2)	74.5(39.2)

BMI indicates body mass index; CVH, cardiovascular health; HCHS/SOL, Hispanic Community Health Study/Study of Latinos; HEI 2010, Healthy Eating Index 2010; LE8, Life’s Essential 8; and SEP, socioeconomic position.

* Socioeconomic mobility was classified into 4 categories: stable low (lower grandparents’ and caregivers’ SEP), downward (higher grandparents’ SEP and lower caregivers’ SEP), upward (lower grandparents’ SEP and higher caregivers’ SEP), and stable high (higher grandparents’ and caregivers’ SEP).

^†^ HEI‐2010 score (continuous [range=0–110]) with higher scores indicating better diet quality.

^‡^ LE8 score and each component (continuous [range=0–100]) with higher scores indicating better CVH.

**Table 2:** Characteristics of Offspring According to Intergenerational Socioeconomic Mobility: SOL Youth

Characteristics	Socioeconomic mobility†				Total*
	Stable high	Stable low	Downward	Upward	
No.	37.2	14.1	15.2	33.6	100.0
Age, y	11.8 (0.2)	12.7 (0.3)	12.3 (0.3)	12.0 (0.2)	12.1 (0.1)
Age group, %					
8–11 y	47.6	32.1	39.3	44.1	552 (43.0)
12–16 y	52.4	67.9	60.7	55.9	665 (57.0)
Sex, %					
Female	49.3	52.6	50.1	48.1	617 (49.5)
Male	50.7	47.4	49.9	51.9	600 (50.5)
Nativity, %					
Foreign‐born	22.7	25.8	15.2	20.2	269 (21.2)
Born in the 50 US states	77.3	74.2	84.8	79.8	938 (78.8)
Hispanic background, %					
Cuban	8.4	1.6	2.3	4.4	79 (5.2)
Central or South American	9.7	4.7	13.2	9.2	135 (9.3)
Dominican	15.9	5.8	14.0	12.0	137 (12.9)
Mexican	33.7	71.2	30.6	63.7	554 (48.7)
Puerto Rican	11.2	6.6	18.3	4.3	104 (9.3)
More than 1/other	16.9	7.7	19.5	5.2	129 (12.1)
Non‐Hispanic	4.2	2.4	2.1	1.3	38 (2.6)
Diet quality (HEI‐2010)‡	54.3 (0.8)	54.6 (1.2)	51.8 (1.4)	54.3 (0.8)	54.0 (0.5)
Total score LE8, %					
Low CVH (<50)	0.5	0.4	1.3	0.8	8 (0.7)
Moderate CVH (≥50 to <80)	72.9	83.1	82.6	78.5	803 (77.8)
High CVH (≥80)	26.6	16.5	16.1	20.7	223 (21.5)
Total score LE8§	72.9 (0.5)	69.6 (1.1)	69.9 (1.0)	72.6 (0.6)	71.9 (0.4)
Blood pressure score	98.3 (0.5)	95.4 (2.3)	97.8 (1.0)	97.7 (0.7)	97.6 (0.5)
Blood glucose score	97.3 (0.6)	94.7 (1.5)	93.7 (1.6)	94.8 (0.9)	95.5 (0.5)
Blood lipids score	73.8 (1.7)	76.3 (2.7)	68.4 (3.2)	73.7 (1.8)	73.3 (1.1)
BMI score	75.0 (2.0)	67.6 (3.6)	70.7 (3.0)	73.2 (2.1)	72.7 (1.2)
Physical activity score	21.9 (0.3)	21.6 (0.5)	23.2 (0.7)	21.3 (0.3)	21.9 (0.2)
Diet score	42.2 (1.9)	40.7 (3.0)	36.4 (3.2)	42.9 (2.0)	41.3 (1.2)
Sleep score	84.0 (1.5)	79.4 (3.0)	82.5 (2.3)	84.2 (1.5)	83.2 (0.9)
Nicotine exposure score	92.8 (0.9)	83.5 (3.5)	84.6 (3.0)	91.7 (1.1)	89.9 (0.9)

BMI indicates body mass index; CVH, cardiovascular health; HEI 2010, Healthy Eating Index 2010; LE8, Life’s Essential 8; SEP, socioeconomic position; and SOL Youth, Hispanic Community Children’s Health Study/Study of Latino Youth.

* Weighted mean (standard error) or number (percentage).

^†^ Socioeconomic mobility was classified into 4 categories: stable low (lower grandparents’ and caregivers’ SEP), downward (higher grandparents’ SEP and lower caregivers’ SEP), upward (lower grandparents’ SEP and higher caregivers’ SEP), and stable high (higher grandparents’ and caregivers’ SEP).

^‡^ HEI‐2010 score (continuous [range=0–110]) with higher scores indicating better diet quality.

^§^ LE8 score (continuous [range=0–100]) with higher scores indicating better CVH.

### Socioeconomic Mobility and Caregivers’ CVH

**Figure 2:**
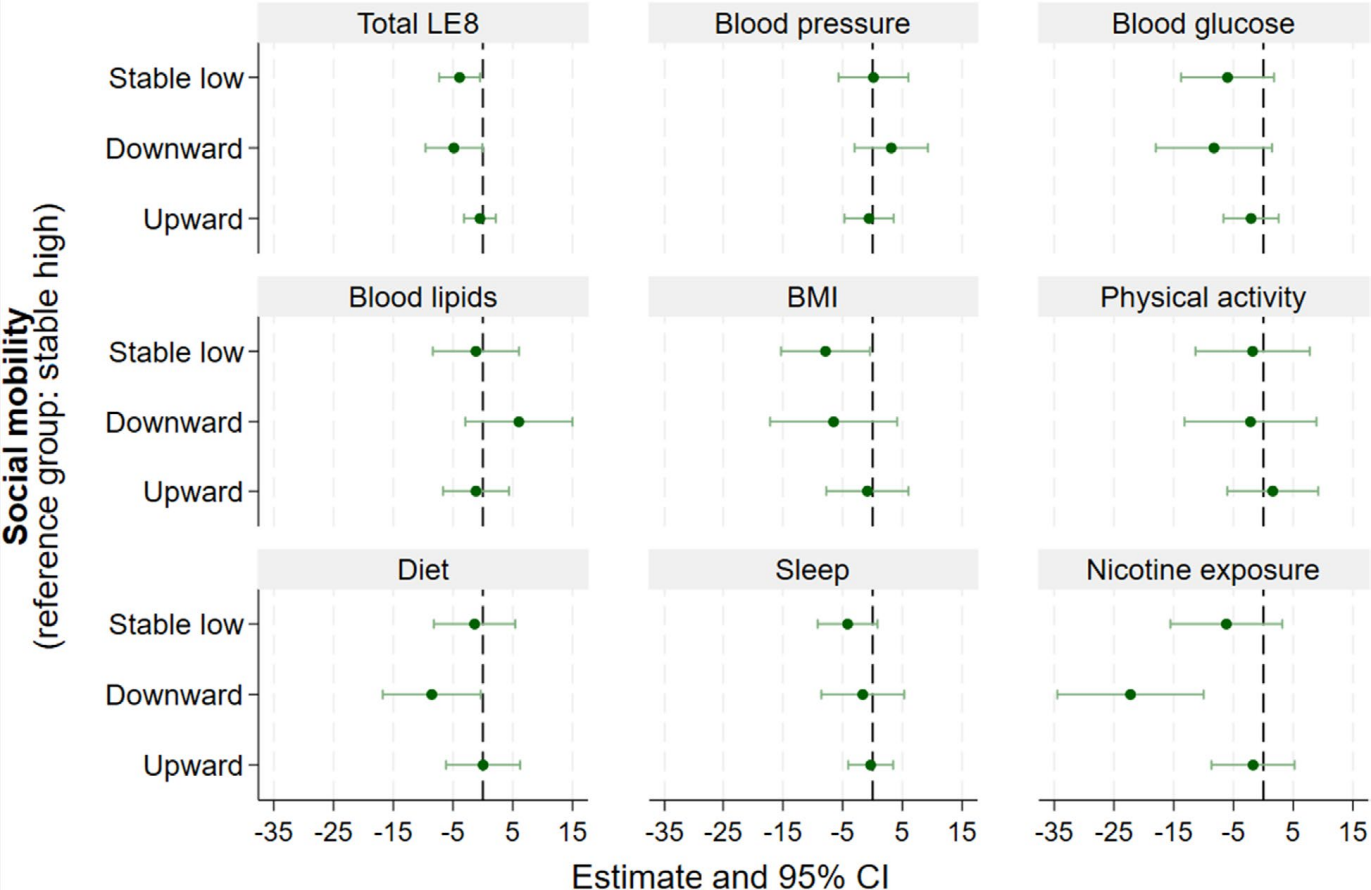
Adjusted mean differences between socioeconomic mobility in caregiver’s total LE8 and its metrics scores. BMI indicates body mass index; and LE8, Life’s Essential 8.

Lower total LE8 scores were observed in caregivers in the stable low (β=−3.91 [95% CI, −7.33 to −0.49]) and downward (β=−4.85 [95% CI, −9.61 to −0.09]) mobility groups compared with those in the stable high group (Figure 2 and Table S2). Caregivers with lower BMI scores were in the stable low group (β=−7.87 [95% CI, −15.31 to −0.43]) compared with those in the stable high group. Caregivers with lower diet (β=−8.57 [95% CI, −16.75 to −0.38]) and nicotine exposure (β=−22.23 [95% CI, −34.48 to −9.98]) scores were in the downward group compared with those in the stable high group.

### Socioeconomic Mobility and Youth’s CVH

**Figure 3:**
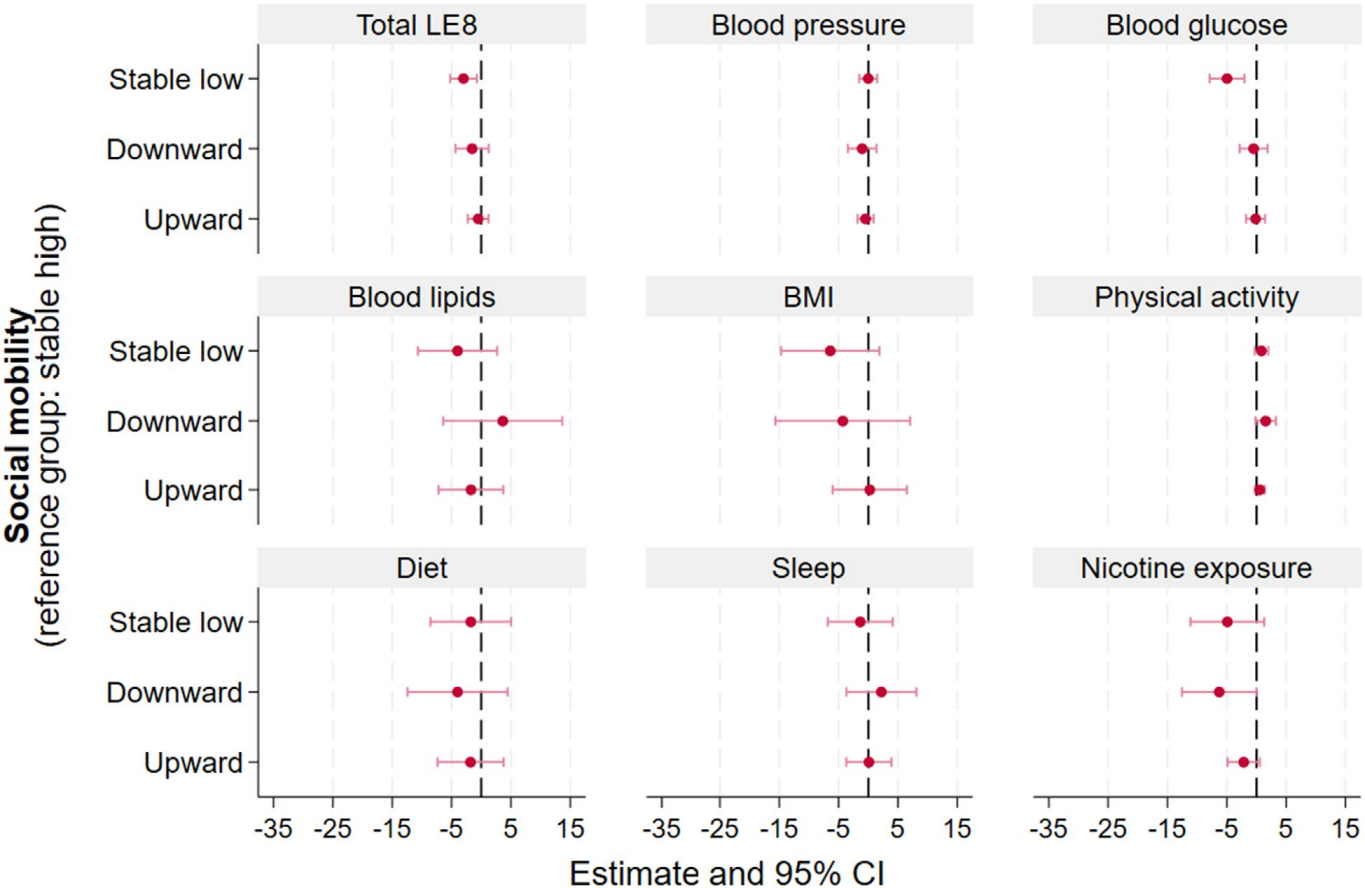
Adjusted mean differences between socioeconomic mobility in youth total LE8 and its metrics scores. BMI indicates body mass index; and LE8, Life’s Essential 8.

We observed lower total LE8 (β=−3.00 [95% CI, −5.24 to −0.75]) and glucose (β=−4.96 [95% CI, −7.89 to −2.04]) scores in youth whose caregivers had stable low socioeconomic mobility compared with those whose caregivers had stable high mobility (Figure 3 and Table S3). We additionally adjusted for parental total LE8 and parental glucose scores, with similar results (Table S3).

We identified some interactions with youth’s sex and age. When comparing the effects of socioeconomic mobility in female versus male youth, there was a significant interaction for lower total LE8 (β_interaction_=−4.80 [95% CI, −9.21 to −0.38]) and blood glucose (β_interaction_=−10.29 [95% CI, −16.75 to −3.83]) scores for the stable low versus stable high group. This negative association was more pronounced in male participants (Table S4).

We also observed lower total LE8 (β_interaction_=−5.09 [95% CI, −9.36 to −0.82]) and lower nicotine exposure (β_interaction_=−8.81 [95% CI, −16.92 to −0.70)] scores for the stable low group versus the stable high group when we compared younger (aged <12 years) with older (aged ≥12 years) youth. This negative association was more pronounced in older youth (Table S5).

### Sensitivity Analysis

In the sensitivity analysis, the significance and estimates of the associations of life socioeconomic mobility with parents’ and youth’s CVH remained similar when analyses were repeated in the subset of caregivers who were biological parents and in the complete cases data set (without imputation). In these sensitivity analyses, we observed lower CVH scores for caregivers and youth who were in the stable low SEP group compared with the stable high SEP group (Tables S6 through S9).

## DISCUSSION

In this study, we found that the prevalence of high CVH (score ≥80) was low in Hispanic/Latino caregivers and youth. Data from NHANES (2013–2018) also found a low prevalences of high CVH among US children and adult populations.^41^ Furthermore, intergenerational socioeconomic mobility was associated with CVH in Hispanic/Latino caregivers and youth. Caregivers in the stable low SEP and downward mobility groups had worse CVH (total LE8 score) than those in the stable high SEP group. Caregivers in the stable low SEP group had lower BMI scores and those in the downward group had lower diet and nicotine exposure scores than those in the stable high SEP group. We found worse overall CVH (total LE8 scores) for youth in the stable low SEP group than those in the stable high SEP group, with lower scores among male and older (aged ≥12 years) youth. Lower glucose scores were also observed for youth in the stable low SEP group compared with the stable high group. In addition, the results for youth overall CVH and glucose scores remained similar after adjusting the models for parental total LE8 and parental glucose scores, respectively.

Contrary to our findings, a previous study using data from the 2 US cohorts (Add Health [National Longitudinal Study of Adolescent to Adult Health] and MINDUS [Maternal and Infant Neurodevelopment Study]) showed that adults in the upward mobility group had worse CVH (defined using metabolic syndrome rates) than those in the stable high mobility group.^42^ We found a higher mean of overall CVH (total LE8 scores) when we compared caregivers in the upward mobility group with those in the stable high mobility group, but the differences were not statistically significant. These differences in findings between studies could be related to a broader definition of CVH in our study, using AHA LE8 metrics rather than the metabolic syndrome, which was used in the Add Health and MINDUS cohorts. In addition, caregivers in the downward mobility group had lower scores for overall CVH, diet, and nicotine exposure than those in the stable high SEP group.

We found some interaction regarding youth’s age and sex when we evaluated the association of caregivers’ socioeconomic mobility and youth’s CVH. Data from NHANES showed lower CVH scores for males and older youth.^41^, ^43^ Previous studies conducted in Brazil, China, and Mexico reported better CVH scores among females than male adolescents.^44^, ^45^, ^46^ A worse CVH was also observed among older (≥15 years) than in younger (<15 years) adolescents in the HELENA (Healthy Lifestyle in Europe by Nutrition in Adolescence) study, a cross‐sectional study conducted in 9 European countries.^47^ These findings may be related to differences in behavioral and environmental exposures according to sex and age.^48^ For example, girls tend to be more subject to parental control than boys, which may impact their behaviors.^49^ In addition, adolescents become more influenced by their social environment than by their family members as they get older.^50^, ^51^ It is important to highlight that these previous studies showed differences in youth’s CVH according to sex and age, but they differ from ours as they did not evaluate how age and sex could modify the relationship between socioeconomic mobility and CVH.

Our findings highlight the low prevalence of high CVH among Hispanic/Latino caregivers and youth. Data from FHS (Framingham Heart Study; original and offspring cohorts) showed similar trends of high CVH among parents and adult offspring, with only a small proportion of them achieving high CVH during 53 years of follow‐up.^52^ Previous studies focused on the transmission of CVH risk factors from parents to offspring and proposed 3 main mechanisms: genetics, in utero environment, and shared postnatal environment.^53^, ^54^, ^55^ However, we still do not fully understand the long‐term effect of socioeconomic adversity on CVH among generations, particularly in disadvantaged populations.

The environment is a potential pathway through which SEP and health disadvantages are transmitted from parents to their offspring.^56^ Caregivers at low SEP, compared with higher SEP, may live in less healthy environments and have greater exposure to ethnic or racial discrimination, which may limit opportunities for healthy food, places to exercise, and well‐being.^57^, ^58^, ^59^ These inequalities can start early^60^, ^61^ and persist throughout childhood. The Developmental Origins of Health and Disease (DOHaD) theory, proposed by Barker, explains that fetal exposure (eg, to maternal poor nutrition and stress) could alter offspring’s neurodevelopment, with later effects on child health and development, including cardiovascular diseases.^62^ After birth, the shared environment continues to contribute to inequalities in child and adolescent health and development, which may impact educational attainment.^63^ Individuals with lower levels of education will have more difficulties in achieving job security and higher incomes.^64^ Therefore, cumulative socioeconomic and health disadvantages can be transmitted, resulting in worse health for the next generation.^56^, ^64^, ^65^

Recently, Houweling and Grunberger (2024) proposed a framework to explain adult health inequalities through 3 pathways: the intergenerational transmission of SEP, the intergenerational transmission of health problems, and the intergenerational transmission of SEP and health.^66^ Previous findings from HCHS/SOL showed that high life‐course SEP (generation 1 and generation 2) was related to better CVH among adults (generation 2).^9^ In addition, findings from SOL Youth showed that Hispanic/Latino youth shared patterns of obesity and cardiovascular risk factors with their parents.^67^ The present study extends these findings by examining how changes in SEP from the youth grandparents (generation 1) to youth parents (generation 2) impact both parents’ (generation 2) and youth’s (generation 3) CVH, highlighting the transmission of SEP and health across the generations.

### Strengths, Limitations, and Future Policies

This study has several strengths, including a diverse sample with information from 3 generations (grandparents, parents, and youth) of persons of Hispanic/Latino background. Previous multigenerational cohorts tend not to include disadvantaged and minoritized groups. Second, the study assessed how changes in SEP across 2 generations (grandparents to parents) can impact both the CVH of the second (parents) and the third (offspring) generation. Exploring this intergenerational transmission is particularly important when we consider the high socioeconomic disadvantage faced by the Hispanic/Latino population living in the United States. The current study also has some limitations. SOL Youth is not representative of the overall Hispanic/Latino youth population in the United States, although communities enrolled in the study were among the US metropolitan areas with the largest concentrations of Hispanic/Latino populations. Even with our cross‐sectional design, a longitudinal component is inherent to the socioeconomic mobility variable (grandparents’ SEP to parents’ SEP); however, we do not have repeated measures of CVH in SOL Youth, which prevents us from assessing changes in CVH over time. The 4 behavior metrics (diet, physical activity, sleep, and nicotine exposure) were self‐reported by youth and their caregivers; thus, we cannot eliminate the possibility of recall bias. We used grandparent education as the only dimension to characterize grandparents’ SEP due to the unavailability of other relevant measures, such as grandparents’ income. This constrained our ability to capture a more comprehensive definition of grandparent SEP. It is important to interpret some results with caution, particularly the categories in which we have a restricted sample size (eg, the downward mobility group). Finally, our data sets do not allow us to compare our findings in the Hispanic/Latino population with other ethnic groups living in the United States and measure whether this association was driven by ethnicity.

Breaking the intergenerational transmission of disadvantages requires multisectoral policies that target these structural disadvantages.^56^, ^66^, ^68^, ^69^, ^70^ Policies focused on improving material conditions, such as supplemental income, job training, quality education access, and affordable housing initiatives, may help reduce food insecurity and exposure to environmental and psychological stress. Policies that expand health care access, such as community‐based health programs or services, may help reduce the barriers that many immigrant families face in accessing affordable health care. However, we believe the integration of these universal policies with a focus on interventions across generations, combining benefits for grandparents, parents, and offspring, is essential to interrupt the disadvantage cycle. Programs that target more than 1 generation (eg, parental job training combined with affordable childcare or quality education for their children) can improve the family’s well‐being and reduce the burden of CVH across generations. Research can inform the development of evidence‐based policies aimed at promoting better health for high‐risk groups. For example, our findings showed that male and older youth whose parents endured social disadvantages had worse CVH. Male teenagers may benefit from policies that integrate school and community‐based programs, while also actively engaging them in the design and evaluation of these interventions.

## CONCLUSIONS

Among Hispanic/Latino individuals living in the United States, the intergenerational persistence of low SEP was associated with worse CVH in both caregivers and youth compared with those remaining in high SEP. This finding supports our initial hypothesis that enduring socioeconomic disadvantages could be associated with worse CVH across generations. The transfer of the wealth gap, limited opportunities for access to quality education and health care, and food insecurity are some of the potential mechanisms that may explain the association of enduring SEP and worse CVH across generations. Future policies focused on these structural interventions to alleviate economic disadvantages may help to address these disparities in CVH. In addition, future research is needed to better understand the critical periods (age stages) where interventions can have a lasting impact.

## Sources of Funding

SOL Youth was supported by grant R01HL102130 from the National Heart, Lung, and Blood Institute (NHLBI). The children in SOL Youth are drawn from the study of adults, HCHS/SOL. HCHS/SOL is a collaborative study supported by contracts from the NHLBI to the University of North Carolina (HHSN268201300001I/N01‐HC‐65233), University of Miami (HHSN268201300004I/N01‐HC‐65234), Albert Einstein College of Medicine (HHSN 268201300002I/N01‐HC‐65235), University of Illinois at Chicago (HHSN268201300003I/N01‐HC‐65236 Northwestern University), and San Diego State University (HHSN268201300005I/N01‐HC‐65237). The following institutes/centers/offices have contributed to HCHS/SOL through a transfer of funds to the NHLBI: National Institute on Minority Health and Health Disparities, National Institute on Deafness and Other Communication Disorders, National Institute of Dental and Craniofacial Research, National Institute of Diabetes and Digestive and Kidney Diseases, National Institute of Neurological Disorders and Stroke, and National Institutes of Health. Additional support was provided by grant RF1AG077639 and the Life Course Methodology Core at Albert Einstein College of Medicine and the New York Regional Center for Diabetes Translation Research (P30 DK111022‐8786 and P30 DK111022) through funds from the National Institute of Diabetes and Digestive and Kidney Diseases; and Mentored Research Scientist Development Award K01 (HL150406) through funds from the NIH and the NHLBI.

## Disclosures

The authors have no conflicts of interest relevant to this article to disclose.

## Supporting information

Tables S1–S9
