# Supplementary material for: Intergenerational Socioeconomic Mobility and Cardiovascular Health Among Hispanic/Latino Youth and Caregivers Living in the United States
Source: J Am Heart Assoc. 2025 Nov 26;14(23):e042972. doi: 10.1161/JAHA.125.042972 (PMC12748529; doi:10.1161/JAHA.125.042972)
Supplement: Supplementary file 1 — Tables S1–S9 [file JAH3-14-e042972-s001.pdf]

Supplemental Material

Table S1. Scoring system for each Life's Essential 8 metric for caregivers and youth in HCHS/SOL and SOL-youth.

	Caregivers		Youth	
**Metric**	**Category**	**Score**	**Category**	**Score**
Diet:	≥P95th	100	≥P95th	100
	P75th–P94th	80	P75th–P94th	80
(based on HEI-2010)	P50th–P74th	50	P50th–P74th	50
	P25th–P49th	25	P25th–P49th	25
	P1st–P24th	0	P1st–P24th	0
Physical Activity:	≥150	100	≥420	100
	120–149	90	360–419	90
(moderate or vigorous minutes/	90–119	80	300–359	80
per week)	60–89	60	240–299	60
	30–59	40	120–239	40
	1–29	20	1–119	20
	0	0	0	0
Nicotine exposure:	Never smoker	100	Never tried	100
	Former smoker, quit ≥5 y	75	Tried any nicotine product, but>30 d ago	50
(Combustible tobacco use or	Former smoker, quit 1–<5 y	50	Current combustible use (any	0
secondhand smoke exposure)	Former smoker, quit <1 y	25	within 30 d)	
	Current smoker	0		
	Subtract 20 points (unless score is 0) for living with active indoor smoker in home		Subtract 20 points (unless score is 0) for living with active indoor smoker in home	
Sleep Health:	7 – <9	100	Age-appropriate optimal range	100
	9 – <10	90	(6y-12y: 9 to12 h and 13y-18y: 8 to 10 h)	
(Self-reported average	6 – <7	70	<1 h above optimal range	90
hours of sleep per night)	5 – <6 or ≥ 10	40	<1 h below optimal range	70
	4 – <5	20	1–<2 h below or ≥1 h above optimal	40
	<4	0	2–<3 h below optimal range	20
			≥3 h below optimal range	0
BMI:	<25	100	P5th– <P85th	100
	25.0–29.9	70	P85th– <P95th	70
(kg/m^2)	30.0–34.9	30	P95th– <120% of the P95th	30
	35.0–39.9	15	120% of the P95th– <140% of the P95th	15
	≥40.0	0	≥140% of the P95th	0
Blood lipids	<130	100	<100	100
	130–159	60	100–119	60
(Non–HDL cholesterol	160–189	40	120–144	40
(mg/dL))	190–219	20	145–189	20

	≥220<br>If drug-treated level, subtract 20 points	0		≥190<br>If drug-treated level, subtract 20 points	0
Blood glucose<br><br>[Fasting blood glucose (FBG) (mg/dL) or HbA1c (%)]	No history of diabetes and FBG <100 (or HbA1c <5.7)<br>No diabetes and FBG 100–125 (or HbA1c 5.7–6.4) (prediabetes)<br>Diabetes with HbA1c <7.0<br>Diabetes with HbA1c 7.0–7.9<br>Diabetes with HbA1c 8.0–8.9<br>Diabetes with Hb A1c 9.0–9.9<br>Diabetes with HbA1c ≥10.0	100<br><br>60<br><br>40<br>30<br>20<br>10<br>0		No history of diabetes and FBG <100 (or HbA1c < 5.7)<br>No diabetes and FBG 100–125 (or HbA1c 5.7–6.4)<br>(prediabetes)<br>Diabetes with HbA1c <7.0<br>Diabetes with HbA1c 7.0–7.9<br>Diabetes with HbA1c 8.0–8.9<br>Diabetes with Hb A1c 9.0–9.9<br>Diabetes with HbA1c ≥10.0	100<br><br>60<br><br>40<br>30<br>20<br>10<br>0
Blood Pressure<br><br>Systolic and diastolic BPs (mm Hg)	<120/<80 (optimal)<br>120–129/<80 (elevated)<br>130–139 or 80–89 (stage 1 hypertension)<br>140–159 or 90–99<br>≥160 or ≥100<br><br>Subtract 20 points if treated level	100<br>75<br>50<br>25<br>0		**Systolic and diastolic BP (mm Hg) percentiles for age through 12 y. For age≥13 y, use adult scoring.**<br>Optimal (<90th percentile)<br>Elevated (≥P90th–<P95th or ≥120/80 mm Hg to<P95th, whichever is lower)<br>Stage 1 hypertension (≥P95th– <P95th+12 mm Hg,or 130/80 to 139/89 mm Hg, whichever is lower)<br>Stage 2 hypertension (≥P95th+12 mm Hg, or≥140/90 mm Hg, whichever is lower)<br>Systolic BP ≥160 or ≥P95th+30 mm Hg systolic BP, whichever is lower; and/or diastolic BP ≥100 or ≥P95th+20 mm Hg diastolic BP<br><br>Subtract 20 points if treated level	 <br>100<br>75<br><br>50<br><br><br>25<br><br>0

Table S2. Association between socioeconomic mobility and caregivers' cardiovascular health, imputing missing data.

	Stable Low		Downward		Upward	
	β	(CI 95%)	β	(CI 95%)	β	(CI 95%)
Total score (n=939)[1]	**-3.91**	**[-7.33; -0.49]***	**-4.85**	**[-9.61; -0.09]***	-0.50	[-3.16; 2.16]
Blood pressure score (n=976)[1]	0.15	[-5.70; 6.01]	3.13	[-3.01; 9.27]	-0.58	[-4.69; 3.54]
Blood glucose score (n=976)[1]	-5.99	[-13.76; 1.78]	-8.27	[-18.00; 1.46]	-2.06	[-6.68; 2.56]
Blood lipid score (n=977)[1]	-1.17	[-8.38; 6.05]	6.02	[-2.94; 14.98]	-1.16	[-6.69; 4.37]
BMI score (n=976)[1]	**-7.87**	**[-15.31; -0.43]***	-6.51	[-17.15; 4.13]	-0.86	[-7.74; 6.02]
Physical activity score (n=977)[1]	-1.80	[-11.36; 7.76]	-2.15	[-13.19; 8.89]	1.58	[-6.03; 9.18]
Diet score (n=972)[1]	-1.40	[-8.20; 5.41]	**-8.57**	**[-16.75; -0.38]***	0.03	[-6.17; 6.23]
Sleep score (n=945)[1]	-4.18	[-9.19; 0.83]	-1.63	[-8.57; 5.30]	-0.32	[-4.08; 3.44]
Nicotine exposure score (n=975)[1]	-6.19	[-15.55; 3.18]	**-22.23**	**[-34.48; -9.98]***	-1.72	[-8.68; 5.24]

* p<.05

[1]Adjusted for caregivers' age, sex, U.S. nativity, Hispanic/Latino background, and field center.

Table S3. Association between socioeconomic mobility and youth cardiovascular health, imputing missing data.

	Stable Low		Downward		Upward	
	β	(CI 95%)	β	(CI 95%)	β	(CI 95%)
**Total score**						
Model 1 (n=1,236)[1]	**-3.00**	**[-5.24; -0.75]***	-1.55	[-4.35; 1.25]	-0.53	[-2.27; 1.22]
Model 2 (n=1,157)[2]	**-2.79**	**[-5.15; -0.44]***	-1.71	[-4.52; 1.10]	-0.56	[-2.21;1.08]
**Blood pressure score**						
Model 1 (n=1,368)[1]	-0.01	[-1.52; 1.50]	-1.02	[-3.44; 1.39]	-0.46	[-1.83; 0.90]
Model 2 (n=1,328)[3]	-0.02	[-1.53; 1.49]	-1.04	[-3.48; 1.39]	-0.47	[-1.84; 0.90]
**Blood glucose score**						
Model 1 (n=1,314)[1]	**-4.96**	**[-7.89; -2.04]***	-0.49	[-2.85; 1.87]	-0.16	[-1.76; 1.45]
Model 2 (n=1,264)[4]	**-4.94**	**[-7.89; -1.98]***	-0.45	[-2.84; 1.93]	-0.14	[-1.75; 1.46]
**Blood lipid score**						
Model 1 (n=1,323)[1]	-4.00	[-10.67; 2.67]	3.62	[-6.41; 13.65]	-1.74	[-7.19; 3.70]
Model 2 (n=1,268)[5]	-3.68	[-10.23; 2.87]	1.35	[-7.87; 10.57]	-1.15	[-6.52; 4.21]
**BMI score**						
Model 1 (n=1,334)[1]	-6.41	[-14.69; 1.88]	-4.29	[-15.64; 7.07]	0.26	[-6.00; 6.51]
Model 2 (n=1,295)[6]	-4.76	[-12.63; 3.12]	-2.64	[-12.32; 7.04]	0.14	[-5.96; 6.24]
**Physical activity score**						
Model 1 (n=1,366)[1]	0.84	[-0.31; 1.98]	1.55	[-0.18; 3.28]	0.56	[-0.29; 1.40]
Model 2 (n=1,327)[7]	0.85	[-0.30; 2.00]	1.56	[-0.17; 3.29]	0.56	[-0.29; 1.41]
**Diet score**						
Model 1 (n=1,360)[1]	-1.76	[-8.56; 5.03]	-4.00	[-12.44; 4.44]	-1.81	[-7.36; 3.75]
Model 2 (n=1,315)[8]	-1.90	[-8.63; 4.82]	-4.89	[-13.30; 3.52]	-1.80	[-7.20; 3.60]
**Sleep score**						
Model 1 (n=1,340)[1]	-1.35	[-6.80; 4.10]	2.21	[-3.69; 8.10]	0.10	[-3.70; 3.90]
Model 2 (n=1,259)[9]	-0.86	[-6.40; 4.68]	1.45	[-4.66; 7.56]	-0.26	[-4.16; 3.64]
**Nicotine exposure score**						
Model 1 (n=1,360)[1]	-4.91	[-11.12; 1.30]	-6.27	[-12.55; 0.00]	-2.16	[-4.88; 0.57]
Model 2 (n=1,318)[10]	-4.72	[-11.09; 1.64]	-5.89	[-12.59; 0.81]	-2.22	[-4.83; 0.40]

* $p<.05$
[1]Adjusted for youth age, sex, U.S. nativity, Hispanic/Latino background, and field center.
[2]Adjusted for youth age, sex, U.S. nativity, Hispanic/Latino background, field center, and caregiver's total score (LE-8).
[3]Adjusted for youth age, sex, U.S. nativity, Hispanic/Latino background, field center, and caregiver's blood pressure score.
[4]Adjusted for youth age, sex, U.S. nativity, Hispanic/Latino background, field center, and caregiver's glucose score.
[5]Adjusted for youth age, sex, U.S. nativity, Hispanic/Latino background, field center, and caregiver's lipid score.
[6]Adjusted for youth age, sex, U.S. nativity, Hispanic/Latino background, field center, and caregiver's BMI score.
[7]Adjusted for youth age, sex, U.S. nativity, Hispanic/Latino background, field center, and caregiver's physical activity score.
[8]Adjusted for youth age, sex, U.S. nativity, Hispanic/Latino background, field center, and caregiver's diet score.
[9]Adjusted for youth age, sex, U.S. nativity, Hispanic/Latino background, field center, and caregiver's sleep score.
[10]Adjusted for youth age, sex, U.S. nativity, Hispanic/Latino background, field center, and caregiver's nicotine exposure score.

Table S4. Association between socioeconomic mobility and youth cardiovascular health by sex.

	β	(CI 95%)
**Total score (n=1,236)**		
Socioeconomic mobility # youth sex		
Stable low # Male	**-4.80**	**[-9.21; -0.38]***
Downward # Male	-0.99	[-6.38; 4.39]
Upward # Male	-3.66	[-7.30; -0.02]
**Blood pressure score (n=1,368)**		
Socioeconomic mobility # youth sex		
Stable low # Male	-0.08	[-3.66; 3.50]
Downward # Male	1.40	[-3.43; 6.24]
Upward # Male	0.08	[-2.73; 2.89]
**Blood glucose score(n=1,302)**		
Socioeconomic mobility # youth sex		
Stable low # Male	**-10.29**	**[-16.75; -3.83]***
Downward # Male	0.06	[-3.67; 3.79]
Upward # Male	-2.84	[-5.85; 0.17]
**Blood lipid score(n=1,323)**		
Socioeconomic mobility # youth sex		
Stable low # Male	-12.92	[-26.39; 0.56]
Downward # Male	-3.32	[-16.37; 9.72]
Upward # Male	-6.71	[-16.53; 3.11]
**BMI score(n=1,334)**		
Socioeconomic mobility # youth sex		
Stable low # Male	-11.85	[-26.43; 2.74]
Downward # Male	-1.43	[-16.44; 13.57]
Upward # Male	-8.90	[-19.68; 1.89]
**Physical activity score (n=1,366)**		
Socioeconomic mobility # youth sex		
Stable low # Male	-0.56	[-2.56; 1.43]
Downward # Male	-0.79	[-3.96; 2.39]
Upward # Male	-0.32	[-1.92; 1.28]
**Diet score(n=1,360)**		
Socioeconomic mobility # youth sex		
Stable low # Male	-6.99	[-18.38; 4.40]
Downward # Male	1.88	[-13.65; 17.41]
Upward # Male	-7.39	[-18.41; 3.62]
**Sleep score(n=1,340)**		
Socioeconomic mobility # youth sex		
Stable low # Male	6.42	[-4.01; 16.85]
Downward # Male	10.24	[-5.31; 25.79]

Upward # Male	6.18	[-2.07; 14.44]
**Nicotine exposure score(n=1,360)**		
Socioeconomic mobility # youth sex		
Stable low # Male	-0.04	[-9.07; 8.99]
Downward # Male	-10.74	[-25.71; 4.24]
Upward # Male	-4.06	[-8.68; 0.56]

* p<.05

[1]Adjusted for youth age, U.S. nativity, Hispanic/Latino background, and field center.

Table S5. Association between socioeconomic mobility and youth cardiovascular health by age (<12y or 12y or more).

	β	(CI 95%)
**Total score (n=1,236)[1]**		
Socioeconomic mobility # youth age		
Stable low # 12y or more	**-5.09**	**[-9.36; -0.82]***
Downward # 12y or more	-0.07	[-5.82; 5.67]
Upward # 12y or more	-2.51	[-6.01; 0.99]
**Blood pressure score (n=1,368)[1]**		
Socioeconomic mobility # youth age		
Stable low # 12y or more	-0.14	[-2.83; 2.56]
Downward # 12y or more	0.93	[-3.92; 5.79]
Upward # 12y or more	-1.65	[-4.34; 1.04]
**Blood glucose score(n=1,302)[1]**		
Socioeconomic mobility # youth age		
Stable low # 12y or more	1.00	[-4.85; 6.84]
Downward # 12y or more	0.95	[-3.81; 5.72]
Upward # 12y or more	-2.70	[-5.83; 0.44]
**Blood lipid score(n=1,323)[1]**		
Socioeconomic mobility # youth age		
Stable low # 12y or more	-11.05	[-24.73; 2.63]
Downward # 12y or more	3.14	[-9.46; 15.74]
Upward # 12y or more	-0.72	[-10.34; 8.90]
**BMI score(n=1,334)[1]**		
Socioeconomic mobility # youth age	-11.09	[-25.96; 3.78]
Stable low # 12y or more	0.40	[-16.19; 17.00]
Downward # 12y or more	-4.63	[-16.61; 7.36]
Upward # 12y or more		
**Physical activity score (n=1,366)[1]**		
Socioeconomic mobility # youth age		
Stable low # 12y or more	-0.56	[-2.91; 1.79]
Downward # 12y or more	-1.80	[-5.52; 1.92]
Upward # 12y or more	-0.43	[-2.11; 1.25]
**Diet score(n=1,360)[1]**		
Socioeconomic mobility # youth age		
Stable low # 12y or more	-5.35	[-18.18; 7.48]
Downward # 12y or more	-6.45	[-25.86; 12.96]
Upward # 12y or more	-5.17	[-16.82; 6.48]
**Sleep score(n=1,340)[1]**		
Socioeconomic mobility # youth age	-2.25	[-12.93; 8.44]
Stable low # 12y or more	7.54	[-8.19; 23.26]
Downward # 12y or more	-2.25	[-12.93; 8.44]
Upward # 12y or more	-3.16	[-11.16; 4.84]
**Nicotine exposure score(n=1,360)[1]**		
Socioeconomic mobility # youth age		
Stable low # 12y or more	**-8.81**	**[-16.92; -0.70]***
Downward # 12y or more	-10.04	[-22.95; 2.86]
Upward # 12y or more	-3.68	[-8.13; 0.77]

*p<.05. [1]Adjusted for youth sex, U.S. nativity, Hispanic/Latino background, and field center.

Table S6. Association between socioeconomic mobility (only biological parents) and caregivers' cardiovascular health.

	Stable Low		Downward		Upward	
	β	(CI 95%)	β	(CI 95%)	β	(CI 95%)
Total score[1]	**-3.86**	**[-7.33; -0.39]***	-4.91	[-9.95; 0.14]	-0.58	[-3.34; 2.19]
Blood pressure score[1]	-0.11	[-6.16; 5.94]	2.54	[-3.99; 9.07]	-0.22	[-4.49; 4.05]
Blood glucose score[1]	-5.07	[-13.19; 3.06]	-9.67	[-19.78; 0.45]	-1.96	[-6.69; 2.77]
Blood lipid score[1]	-0.73	[-8.16; 6.69]	6.67	[-2.70; 16.03]	-0.83	[-6.71; 5.04]
BMI score[1]	**-8.08**	**[-15.80; -0.35]***	-6.34	[-17.64; 4.96]	-1.36	[-8.35; 5.63]
Physical activity score[1]	-1.35	[-11.19; 8.48]	-2.26	[-13.70; 9.18]	1.61	[-6.40; 9.62]
Diet score[1]	-2.15	[-9.20; 4.90]	**-9.13**	**[-17.44; -0.83]***	-1.07	[-7.31; 5.17]
Sleep score[1]	-4.30	[-9.46; 0.86]	-1.68	[-8.89; 5.53]	-0.57	[-4.43; 3.28]
Nicotine exposure score[1]	-6.20	[-15.81; 3.42]	**-21.25**	**[-34.17; -8.33]***	-1.41	[-8.59; 5.77]

* p<.05.
[1]Adjusted for caregivers' age, sex, U.S. nativity, Hispanic/Latino background, and field center.

Table S7. Association between socioeconomic mobility (only biological parents) and youth cardiovascular health.

	Stable Low		Downward		Upward	
	β	(CI 95%)	β	(CI 95%)	β	(CI 95%)
Total score[1]	**-3.19**	**[-5.50; -0.88]***	-1.82	[-4.79; 1.16]	-0.76	[-2.61; 1.08]
Blood pressure score[1]	-0.06	[-1.64; 1.53]	-1.05	[-3.59; 1.49]	-0.62	[-2.08; 0.83]
Blood glucose score[1]	**-5.06**	**[-8.12; -2.00]***	-0.74	[-3.16; 1.67]	0.07	[-1.60; 1.73]
Blood lipid score[1]	-4.37	[-11.35; 2.61]	3.95	[-6.43; 14.33]	-2.00	[-7.77; 3.78]
BMI score[1]	-7.00	[-15.52; 1.51]	-5.35	[-17.22; 6.52]	0.28	[-6.13; 6.70]
Physical activity score[1]	0.72	[-0.49; 1.92]	1.08	[-0.72; 2.88]	0.61	[-0.27; 1.49]
Diet score[1]	-1.98	[-9.04; 5.08]	-3.97	[-12.85; 4.92]	-2.37	[-8.19; 3.45]
Sleep score[1]	-0.60	[-6.14; 4.93]	1.70	[-4.26; 7.66]	-0.41	[-4.37; 3.55]
Nicotine exposure score[1]	-5.49	[-11.97; 0.99]	-6.40	[-13.04; 0.24]	-2.71	[-5.53; 0.11]

* p<.05

[1]Adjusted for youth age, sex, U.S. nativity, Hispanic/Latino background, and field center.

Table S8. Association between socioeconomic mobility and caregivers' cardiovascular health, complete case data.

	Stable Low		Downward		Upward	
	β	(CI 95%)	β	(CI 95%)	β	(CI 95%)
Total score (n=863)[1]	**-3.74**	**[-7.29; -0.20]***	-3.93	[-8.97; 1.12]	-0.43	[-3.16; 2.29]
Blood pressure score (n=900)[1]	-0.40	[-6.67; 5.87]	2.10	[-4.77; 8.96]	-0.79	[-4.96; 3.39]
Blood glucose score (n=900)[1]	-6.56	[-14.85; 1.73]	-6.08	[-16.26; 4.11]	-2.02	[-6.71; 2.67]
Blood lipid score (n=901)[1]	-1.30	[-8.69; 6.08]	6.14	[-3.47; 15.75]	-1.04	[-6.70; 4.61]
BMI score (n=900)[1]	**-8.05**	**[-15.62; -0.48]***	-4.19	[-15.01; 6.63]	-0.38	[-7.26; 6.51]
Physical activity score (n=901)[1]	0.29	[-9.51; 10.09]	0.27	[-11.07; 11.61]	1.74	[-6.02; 9.50]
Diet score (n=896)[1]	-0.65	[-7.59; 6.28]	-7.68	[-16.43; 1.07]	0.15	[-6.27; 6.56]
Sleep score (n=869)[1]	-4.72	[-9.96;0.51]	-1.01	[-7.96; 5.94]	-0.35	[-4.17; 3.48]
Nicotine exposure score (n=899)[1]	-5.18	[-15.18; 4.82]	**-22.91**	**[-35.75; -10.06]***	-1.89	[-9.09; 5.31]

* p<.05

[1]Adjusted for caregivers' age, sex, U.S. nativity, Hispanic/Latino background, and field center.

Table S9. Association between socioeconomic mobility and youth cardiovascular health, complete case data.

	Stable Low		Downward		Upward	
	β	(CI 95%)	β	(CI 95%)	β	(CI 95%)
**Total score**						
Model 1 (n=1,086)[1]	**-3.08**	**[-5.59; -0.58]***	-1.29	[-4.10; 1.52]	-0.18	[-1.94; 1.58]
Model 2 (n=1,042)[2]	**-3.20**	**[-5.84; -0.55]***	-1.88	[-4.79; 1.03]	-0.34	[-2.02; 1.35]
**Blood pressure score**						
Model 1 (n=1,208)[1]	-0.91	[-3.91; 2.09]	-0.83	[-3.23; 1.57]	-0.21	[-1.58; 1.15]
Model 2 (n=1,207)[3]	-0.93	[-3.93; 2.07]	-0.94	[-3.37; 1.49]	-0.21	[-1.58; 1.16]
**Blood glucose score**						
Model 1 (n=1,146)[1]	**-5.54**	**[-8.77; -2.31]***	-0.84	[-3.39; 1.70]	-0.30	[-1.92; 1.32]
Model 2 (n=1,145)[4]	**-5.55**	**[-8.80; -2.30]***	-0.84	[-3.39; 1.71]	-0.29	[-1.92; 1.32]
**Blood lipid score**						
Model 1 (n=1,166)[1]	-2.92	[-9.64; 3.80]	2.10	[-6.96; 11.16]	-0.32	[-5.78; 5.13]
Model 2 (n=1,166)[5]	-2.98	[-9.62; 3.67]	-0.27	[-9.00; 8.46]	0.06	[-5.33; 5.45]
**BMI score**						
Model 1 (n=1,177)[1]	-6.69	[-15.11; 1.73]	-0.06	[-11.47; 11.34]	-0.21	[-6.25; 5.83]
Model 2 (n=1,175)[6]	-5.64	[-13.76; 2.47]	0.45	[-9.30; 10.20]	-0.46	[-6.58; 5.66]
**Physical activity score**						
Model 1 (n=1,206)[1]	0.94	[-0.21; 2.08]	**1.82**	**[0.06; 3.59]***	0.63	[-0.21; 1.46]
Model 2 (n=1,206)[7]	0.95	[-0.20; 2.10]	**1.82**	**[0.06; 3.59]***	0.63	[-0.21; 1.46]
**Diet score**						
Model 1 (n=1,201)[1]	-1.08	[-8.26; 6.09]	-2.94	[-12.40; 6.53]	-0.43	[-6.11;5.25]
Model 2 (n=1,195)[8]	-1.50	[-8.67; 5.66]	-3.76	[-13.10; 5.58]	-0.41	[-5.94;5.12]
**Sleep score**						
Model 1 (n=1,183)[1]	-2.23	[-8.36; 3.90]	2.32	[-4.62; 9.25]	-0.21	[-4.32; 3.90]
Model 2 (n=1,141)[9]	-1.85	[-8.04; 4.35]	1.35	[-5.89; 8.59]	-0.63	[-4.78; 3.51]
**Nicotine exposure score**						
Model 1 (n=1,201)[1]	-6.25	[-14.39; 1.90]	**-9.31**	**[-16.58; -2.03]***	-2.45	[-5.39; 0.50]
Model 2 (n=1,197)[10]	-6.31	[-14.57; 1.95]	**-8.94**	**[-16.55; -1.33]***	-2.58	[-5.43; 0.26]

* p<.05

[1]Adjusted for youth age, sex, U.S. nativity, Hispanic/Latino background, and field center.

[2]Adjusted for youth age, sex, U.S. nativity, Hispanic/Latino background, field center, and caregiver's total score (LE-8).

[3]Adjusted for youth age, sex, U.S. nativity, Hispanic/Latino background, field center, and caregiver's blood pressure score.

[4]Adjusted for youth age, sex, U.S. nativity, Hispanic/Latino background, field center, and caregiver's glucose score.

[5]Adjusted for youth age, sex, U.S. nativity, Hispanic/Latino background, field center, and caregiver's lipid score.

[6]Adjusted for youth age, sex, U.S. nativity, Hispanic/Latino background, field center, and caregiver's BMI score.

[7]Adjusted for youth age, sex, U.S. nativity, Hispanic/Latino background, field center, and caregiver's physical activity score.

[8]Adjusted for youth age, sex, U.S. nativity, Hispanic/Latino background, field center, and caregiver's diet score.

[9]Adjusted for youth age, sex, U.S. nativity, Hispanic/Latino background, field center, and caregiver's sleep score.

[10]Adjusted for youth age, sex, U.S. nativity, Hispanic/Latino background, field center, and caregiver's nicotine exposure score.
